# Notes and News

**Published:** 1891-05-16

**Authors:** 


### Notes and News.

Collected and Communicated.

"St. John of Jerusalem stops the way." This sounds rather flippant, but Catholics will understand; for it is a well-known fact that Cardinal Manning will foster no new scheme till the Hospital of St. John and St. Elizabeth, Great Ormond Street, has got the £8,000 it needs so badly. The Prioress of St. John was one of Miss Nightingale's little band of nurses during the Crimea, and she is anxious to add another fifty beds to the hospital, which shelters, not only the incurable, but the dying—when it has room! It is a truly worthy charity, and we commend its needs to our Catholic readers.

The Royal Sea-bathing Infirmary, Margate, established for the benefit of those suffering from scrofula, celebrated its one hundredth birthday at the Freemasons' Tavern, Great Queen Street, W.C., on Monday, the Right Hon. the Marquis of Lorne, K.T., presiding. It made this festival the opportunity for seeking to establish a "Special Centenary Endowment Fund" of £50,000, by devoting the interest of which solely to maintenance the management hope to some extent to prevent the annual deficit by which the small invested income (some £6,791) is being rapidly eaten up. The expenditure is about £10,000 per annum, while the income comes to about £2,000 less, and this deficiency has already necessitated the closing of two of the wards. This should not be so, seeing that really poor patients have their fees remitted, and that the percentage of cases discharged cured has risen from 23 per cent. in 1886 to 53 per cent. in 1890.

The Committee appointed to consider and report as to the management and constitution of the Kent and Canterbury Hospital have completed their inquiry, which led them to the following conclusions:—That there is no proper and effectual control over the expenditure; that the number of beds is in excess of the present income of the hospital; that patients are admitted whose financial position, and also the chronic nature of whose complaints, render them unfit for admission; and that it is impossible for the Board of Management, meeting once a month, to exercise that close control which is necessary over such an institution. The Committee suggest that the upper wards of the hospital should be closed, and thus reduce the number of beds to 70. Since 1875 legacies amounting to £14,600 have been received by the hospital, the whole of which amount, with the exception of £329 Consols, has been swallowed up in meeting the yearly deficits, and in addition there is a balance of £1,306 15s. 1d. standing against the hospital at the end of last year, and it is feared that if this excess of income over expenditure continues it will, in the course of years, involve the sacrifice of the invested capital. The Committee propose that the future management of the hospital should be placed in the hands of a Hospital Committee, who shall meet every week.

A little institution, which deserves liberal support from the large towns of Yorkshire, is the Seaside Home, Whitby, which was established to relieve the necessities of the sick poor in crowded towns, and to give the women and children of the labouring and mechanic classes the advantages of pure air and sea-bathing. The home is open from the first week in April until the first week in December. 185 sick persons were admitted last year, some of whom remained two or three months in order to perfect their cure. The Home has been hampered by a large bank debt, but last year this was reduced from £652 13s. 6d. to £290 by a very successful bazaar which was held in August.

The annual meeting of the friends of the City of Dublin Hospital was held on April 18th, under the presidency of Lord Justice Fitzgibbon. The report stated that during the year 1,088 patients passed through the wards, and 14,578 patients attended at the out-patient department. The Board have purchased a house adjoining the Hospital, and trust, as soon as funds will permit, to increase the accommodation for patients, and also to improve the arrangements for the resident staff and nurses in the Hospital. There is a deficit of £548 0s. 1d. on the year's working, and this would have been much larger but for legacies received to the amount of £1,188. The income of the Hospital is very inadequate to meet the necessary expenditure.

The Rev. Prebendary Whittington presided at the festival dinner of the City of London Truss Society, on the 28th ult., in the absence of the Lord Mayor through indisposition. He was, without doubt, the right man in the right place. Called upon at only thirty minutes' notice to take the place of the Lord Mayor, he made an able and enthusiastic speech in behalf of the Society, and with apparently a very satisfactory result. Contrary to the experience of many charities, the subscription list shows a steady increase, but so also does the number of patients (and at a greater ratio), and it is a matter of regret that the Society has been compelled to sell out a portion of its funded property. The Secretary read out a list of subscriptions amounting to upwards of £800, which was considerably more than that of any previous year.

The annual court of the Governors of the Royal Mineral Water Hospital, Bath, was held on May 1st. General Mainwaring presided, and there was a large attendance. Before the proceedings began, the Chairman alluded to the serious loss the Hospital had sustained through the death of the senior physician, Dr. Hensley, whose kindness to the sick and suffering would long be remembered. The Registrar submitted the annual report, which stated that the number of applications for admission was 1,495, the average stay of each patient being 43 days. The total receipts from all sources amounted to £5,731 19s. 3d., while the expenditure was £5,227 12s. 2d., leaving a balance at the bank of £506 7s. 1d. The report was unanimously adopted, and the proceedings ended with a vote of thanks to the Chairman.

The Zenana Bible and Medical Mission held its annual meeting in Prince's Hall, on April 29th. Lord Kinnaird was in the chair, and the meeting was well attended. Mr. T. W. Paton, hon. Secretary, read a very satisfactory report, which stated that the contributions to the fund had reached £17,000, an increase of £4,446, and the largest amount received since the foundation of the society in 1852. Two hospitals and five dispensaries are now open, with five lady doctors in attendance. The foundation of a new hospital has been laid at Benares, and another is in the course of erection at Lucknow as a memorial to Lord Kinnaird's mother. Miss Leitch, in an earnest address, appealed to the ladies present to give larger support to the missionary cause. The society called for £30,000, so that its income and its work might be doubled. Subscriptions amounting to £1,087 were announced.

